# A Prize

**Published:** 1866-08

**Authors:** 


### A PRIZE.

At the meeting of the Mississippi Valley Dental Association held in Feb. last, a prize of one hundred dollars was offered, through the Association, by Drs. Morgan, of Nashville, Mc-

Clelland, of Louisville and Cameron, of Cincinnati, for the best essay upon "The Health of the Dentist, the peculiar diseases to which he is liable, and the best means of avoiding them."

A prize of fifty dollars is also offered by Dr. Morgan, through the Central States Dental Association, for the best essay upon the same subject.

The essays presented for these premiums are to be submitted to committees appointed for the purpose, and are to be disposed of according to the regulations made by the Mississippi Valley Dental Association at it meeting in 1865.

We hope these prizes may be the means of calling out something valuable to the profession. It is a subject interesting to every member of the profession. We would request medical journals, so far as practicable, to give this notice an insertion.

